# Bioactivity of Humic Acids Extracted From Shale Ore: Molecular Characterization and Structure-Activity Relationship With Tomato Plant Yield Under Nutritional Stress

**DOI:** 10.3389/fpls.2021.660224

**Published:** 2021-05-26

**Authors:** Hiarhi Monda, Amy M. McKenna, Ryan Fountain, Richard T. Lamar

**Affiliations:** ^1^Humic R&D Lab, Bio Huma Netics, Inc., Gilbert, AZ, United States; ^2^National High Magnetic Field Laboratory, Ion Cyclotron Resonance Facility, Tallahassee, FL, United States

**Keywords:** humic acids, biostimulant activity, nutrient stress, FT-ICR MS of humic substances, reactive oxygen species, redox (bio)geochemistry, antioxidant and prooxidant, quinones and flavonoids

## Abstract

The increasing demands for biostimulants in the agricultural market over the last years have posed the problem of regulating this product category by requiring the industry to make available the information about efficacy and safety, including the explanation of mode of action and the definition of bioactive constituents. In the present study, we tested the biostimulant proprieties of a sedimentary shale ore-extracted humic acid (HA) on Micro Tom tomato plants under increasing nutritional stress and investigated the correlation with the chemical features of HA by means of ultra-high resolution FT-ICR MS, FT-ATR, and ^13^C-NMR. Humic acid application proved effective in alleviating the nutritional stress by improving nutrient use efficiency, with results comparable to the control treatment supplied with higher NPK nutrition. Increased yield (up to +19%) and fruit quality (in the range +10–24%), higher ascorbic acid content and a better root growth were the main parameters affected by HA application. Molecular-level characterization identified the possible chemical drivers of bioactivity, and included flavonoids, quinones, and alkaloids among the most represented molecules, some of which exhibiting antioxidant, pro-oxidant, and antimicrobial activity. The redox effect was discussed as a determinant of the delicate homeostasis balance, capable of triggering plant defense response and eventually inducing a protective priming effect on the plants.

## Introduction

Humic substances (HS) are the major component of natural organic matter (NOM), a complex mixture of organic compounds naturally occurring in soils, water, and sediments (Stevenson, 1994). The current use of the operationally based definition of HS fractions, although originally applied strictly to the soil, has been applied to a variety of different sources (Weber et al., 2018). Soil, peat, ores, sediments, leonardite, lignite, compost, and plant are just some sources out of a possibly longer list, each one performing differently based on its own chemistry. During the last two centuries the knowledge about direct and indirect effects of HS on soil fertility and plant growth has evolved, but the complexity of their constituents and the diversity of each source did not allow the harmonization and standardization of the information accumulated. Conversely, supported by the lack of appropriate technologies and a vague operational definition, ambiguities, and uncertainties about HS origin and chemical structure arised (Kelleher and Simpson, 2006; Kleber and Lehmann, 2019; Olk et al., 2019; Hayes and Swift, 2020). As a consequence, the understanding of their mode of action has been delayed.

However, the biostimulant proprieties of HS have been gradually recognized by the agricultural community that contributed to pushing the market into a rising trend that is expected to increase the global returns at a rate between 9 and 13.4% by 2025 (Meticulous Market Research, 2019; Khillari, 2020). This fast expansion has led to the introduction of new government regulations requiring the elucidation of the mode of action to legitimize the biostimulant industry. A detailed characterization of the chemical composition becomes therefore critical in order to understand the structure-activity relationship and to finally supply farmers with effective products with claims based on science.

Nonetheless, the reduction of agrochemicals footprint and the adoption of an efficient nutrient management need to be implemented to promote a sustainable food production (Vitousek et al., 2009; Foley et al., 2011; Rouphael and Colla, 2018).

Much information has been accumulated regarding the mechanisms by which plants react to HS application (Nardi et al., 1991; Canellas et al., 2002, 2015; Zandonadi et al., 2010; Zanin et al., 2018; Pizzeghello et al., 2020; Olaetxea et al., 2021), as well as their interaction with the rhizospheric microbiome, ultimately leading to enhanced plant development (Puglisi et al., 2009, 2013; Maji et al., 2017; De Hita et al., 2020). However, when moving from short time lab-scale experiments to greenhouse or field experiments where final productivity is measured, impaired results are often reported. Azcona et al. (2011) found that pepper plants treated with HS from composted sludge did not show an improved nutrient uptake or differences in total fruit yield, despite an overall increased biomass produced. Similarly, Pilanal and Kaplan (2003) in a 2-year greenhouse experiment found that foliar application of HA did not affect nutrient uptake in mature strawberry leaves.

According to Rose et al. (2014) HS seem to be more effective when plants are grown under stress. A growing body of literature is accumulating about abiotic stress relief of HS, but little research is available on plants subjected to nutrient stress. Tavares et al. (2019) found that rice plants grown hydroponically and pre-treated with HA showed increased net influx of ${\text{NO}}_{3}^{-}$ after a temporary nitrogen deprivation. The only paper found addressing the nutrient stress under field conditions reported an increased P uptake and yield along with the improvement of the antioxidant defense system in maize plants treated with leonardite HA under P deficiency (Kaya et al., 2020). The mitigation of stress has been linked to the ability of HS to prevent ROS induced oxidative damage by modulation of redox homeostasis (García et al., 2016a; Roomi et al., 2018). However, the role of HS chemical structure in biostimulation is not well understood and requires more investigations because an unequivocal relationship has not been identified, despite previous studies which demonstrated the importance of chemical composition and source in predicting the bioactivity of HS (Aguiar et al., 2013; Martinez-Balmori et al., 2014; Monda et al., 2018).

Relevant advances during the last decades, in elucidating the chemical nature of HS, have been achieved using several different techniques such as NMR, pyrolysis GC-MS, LC-MS, FT-IR, fluorescence spectroscopy, and HP-SEC. However, although assessing the general chemical nature of these materials by being marginally successful, none of these techniques has yielded molecular level information until the breakthrough introduction of ultra-high-resolution techniques. Fourier transform ion cyclotron resonance mass spectrometry in a high magnetic field (FT-ICR MS) has become one of the most important analytical tools for detailed characterization of complex mixtures due to its ultra-high mass resolving effectiveness. To date, extended literature has been produced on the application of FT-ICR MS to NOM and its fractions, mostly in relation to dissolved organic matter (DOM) (Brown and Rice, 2000; Kujawinski, 2002; Stenson et al., 2002; Sleighter and Hatcher, 2007; Remucal et al., 2012; Lv et al., 2016). But, when it comes to the terrestrial soil organic matter (SOM) and its fractions, only a few publications arise (Kramer et al., 2004; Ohno et al., 2010; Piccolo et al., 2010; Ohno and Ohno, 2013; Zherebker et al., 2019), whereas investigations on other sources such as ores or compost are rare. A recent innovative approach has been incorporated in the pipeline of the studies on biologically active metabolites, where molecular formulas obtained by FT-ICR MS were sourced from public online databases with valuable results that helped gathering insights into the chemistry of HS (Fedoros et al., 2018; Orlov et al., 2019; Zhernov et al., 2020).

The objective of the present study was to investigate in detail the chemical features of HA extracted from sedimentary ore with the aim of exploring the potential relationship of chemical function with biostimulant activity, and to evaluate the extent to which the priming effect of HA on tomato plants under nutritional stress was reflected on the yield gains.

## Materials and Methods

### Ore Humic Acids Extraction and Elemental Composition

A sedimentary lignite ore (Idaho, USA), ground to pass a 1,000 μm sieve, was used as the source of HA (IDHA). Isolation of HA was obtained by alkaline extraction according to International Humic Substances Society (IHSS) procedure (Swift, 1996). Purification step through HCl/HF was performed to reduce the mineral ash content (Lamar et al., 2014).

The elemental composition of the purified HA extract was achieved by combustion analysis. Carbon and Nitrogen were determined by catalytic combustion with a Rapid CS Cube combustion analyzer and a Rapid MAX N Exceed combustion analyzer both from Elementar Americas, Inc. (Elementar, Ronkonkoma, NY, USA).

### ESI FT-ICR Mass Spectrometry

Extracted samples were analyzed with a custom-built 9.4 T FT-ICR mass spectrometer at the National High Magnetic Field Laboratory, equipped with a horizontal, 220 mm bore diameter operated at room temperature, and a modular ICR data station (Predator 32) facilitated instrument control, data acquisition, and data analysis (Blakney et al., 2011; Kaiser et al., 2014). A purified HA sample was first dissolved in NH_4_OH (30%), followed by double dilution with MeOH:H_2_O (1:1) to a concentration of 100 mg L^−1^ (Rostad and Leenheer, 2004). The mass spectrum was acquired in negative ionization mode with an introduction flow rate of 0.5 μL min^−1^, ESI needle voltage of−3,000 V, 100 scan accumulation, and 400 ms event length. 100 individual transients of 5.8–6.1 s duration collected for crude extracts were averaged, apodized with a Hanning weight function, and zero-filled once prior to fast Fourier transformation. For all mass spectra, the achieved spectral resolving power approached the theoretical limit over the entire mass range, e.g., average resolving power, m/Δm_50%_, in which Δm_50%_ is mass spectral peak full width at half-maximum peak height was ~1,000,000–1,300,000 for absorption mode at m/z 500 for all mass spectra and processed in absorption mode (Beu et al., 2004; Xian et al., 2010, 2012). Peaks with signal magnitude greater than six times the baseline root-mean-square (RMS) noise level were exported to a peak list. The spectrum was internally calibrated by using known methylene homologous series and molecular formula assignments of the resulting mass spectra considering C_c_H_h_N_n_O_o_S_s_ chemical species (Savory et al., 2011; McKenna et al., 2019). Mass peaks with S/N>6 were processed for formula assignment by using the National High Magnetic Field Laboratory, Ion Cyclotron Resonance Facility PetroOrg^©^ software (Corilo, 2018) by setting the following parameters: ^12^C_1−100_^1^ H_2−200_, ^6^O_2−30_, ^14^N_0−3_, ^32^S_0−3_ with a mass error threshold set at ≤0.5 ppm. Formulae having the least N and S were assigned first (Kujawinski et al., 2009). Generated formulae were filtered by O/C ratio (≤1) and H/C ratio (≤2) according to Koch et al. (2005). The degree of hydrogen and oxygen saturation and molecular heterogeneity were assessed within the assigned formulae and molecular reactivity analyzed based on H/C and O/C ratios by means of a Van Krevelen diagram (Van Krevelen, 1950) whose molecular compositional space was divided into the typical classes of discrete organic biomolecules found in organic matter according to the following rules: (1.5 < H/C < 2; O/C ≤ 0.3) Lipid-like, (1 < H/C < 2.2; 0.1 < O/C < 0.67; *N* ≥ 1) Protein-like, (0.7 < H/C < 1.5; 0.1 < O/C < 0.67) Lignin-like, (H/C >1.5; O/C > 0.67) Carbohydrate-like, (0.2 < H/C < 0.7; O/C ≤ 0.67) CAS Condensed aromatic structures, (0.7 < H/C < 1.5; O/C ≤ 0.1) UHC Unsaturated hydrocarbons (Hockaday et al., 2009). Online databases such as ChEMBL and PubChem were used to tentatively estimate the potential isomeric structures of the most abundant group of molecules identified by FT-ICR MS data. The formulae most represented in each heteroatomic group were matched online and the most common structures selected when similar features were identified. It should be noted that structure identification might not be indicative of the actual isomer configuration.

### ^13^C-CPMAS NMR Spectroscopy

A 300 MHz Bruker Avance spectrometer, equipped with a 4 mm wide-bore MAS probe, was used to run solid-state spectra of the HA sample. Powdered sample was packed into a 4 mm zirconium rotor, stoppered with a Kel-F cap and spun at a rate of 13,000 ±1 Hz. A ^13^C-NMR spectrum was acquired through the Cross-Polarization Magic-Angle-Spinning (CPMAS) technique with the following parameters: 2 s of recycle delay, 1 ms of contact time, 30 ms of acquisition time, and 4,000 scans. The spectrum was processed by using both Bruker Topspin Software (v.2.1, Bruker Biospin, Rheinstetten, Germany) and MestReC NMR Processing Software (v.4.8.6.0, Cambridgesoft, Cambridge, Massachusetts, USA). Integration of the chemical shift was performed as follows: (0–45 ppm) Alkyl-C, (45–60 ppm) Methoxyl-C, (60–95 ppm) O-Alkyl-C, (95–110 ppm) O2-Alkyl-C, (110–145 ppm) Aryl-C, (145–165 ppm) O-Aryl-C, (165–210 ppm) Carbonyl-C. Structural indices that provided additional biochemical characterization were calculated as follows: hydrophobicity index, HB = (0–45 + 110 – 145 + 145 – 165 ppm)/(60 – 110 + 165 – 210 ppm), alkylic ratio, Alk-R = (0 – 45 ppm)/(60 – 110 ppm), lignin ratio, LigR = (45 – 60 ppm)/(145 – 165 ppm), aromaticity index, AI = (110 – 165 ppm)/(0 – 110 + 165 – 210 ppm) (Spaccini and Piccolo, 2007).

### Molecular Mixing Model

Mathematical algorithms of the molecular mixing model (MMM) were used to extract relevant quantitative information from NMR and MS data as described by Baldock et al. (2004) and modified by Hockaday et al. (2009). Briefly, the model uses NMR peak areas to estimate the relative proportion of six components that represent the major biomolecule classes found in natural organic matter to describe the molecular composition of the sample. The six classes correspond to: Carbohydrate, protein, lignin, aliphatic, carbonyl and char. The model is built upon the empirical data obtained for terrestrial and marine environments. The linear combination of the six components allows the model to calculate the best fit to the measured NMR area distribution. As a means of quantitative matching, the MS data obtained by classification of molecular formulae into biochemical categories were used to run the MMM by reverse approach and predict the signal distribution for a ^13^C-CPMAS NMR. In this way, it was possible to compare in a meaningful way the two analytical techniques and assess the degree to which the molecular distribution relates to the elemental composition.

### FT-IR ATR Spectroscopy

An infrared (IR) spectrum was recorded on a Perkin-Elmer Spectrum Two Infrared Spectrometer using an attenuated total reflection (ATR) device equipped with a diamond/ZnSe crystal. About 5 mg HA powder was weighed and put in contact with the crystal by applying a strength of about 150 N on the sample. The spectrum was acquired by using 32 scans with resolution of 4 cm^−1^ from the 4,000 to 400 cm^−1^ region. The sample was analyzed 3 times and the average of these spectra was used for data interpretation.

### Tomato Plant Pot Experiment and Analysis

Tomato seeds (*Solanum lycopersicum* L. cv. Micro-Tom) were surface sterilized in 3% NaClO for 10 min and water rinsed thoroughly before individually sowing in pots containing a mixture of coconut coir and sand (2:1). Plants were grown for three months in a climate-controlled growth chamber set at 28°C with a light/dark cycle of 14/10 h, light intensity set at 300 μmol m^−2^ s^−1^ and relative humidity of 65%. At fifteen days seedlings started receiving nutrition as Hoagland solution with quarter (25), half (50) or full (100) NPK dose and watered at 70% of water holding capacity. Humic acids were added at the pre-plant stage to a concentration of 80 mg C L^−1^. Nutritional dose and HA concentration were selected based on a previous experiment so that a nutritional stress condition was triggered at low nutrient dose (data not shown). A total of six treatments with eight replicates per treatment were arranged in a randomized complete block design. During the experiment plant height was tracked, and chlorophyll content measured by a chlorophyll meter MC-100 (Apogee Instruments, Logan, UT, USA). Chlorophyll fluorescence was determined at noon by using a OS30p+ pulse modulated fluorometer (Opti-Sciences, Hudson, NH, USA) after leaves were subjected to a dark adaptation period of 20 min, followed by the measure of the ratio F_V_/F_0_, were F_V_ is the difference between the maximum and minimum fluorescence and F_0_ is the minimum fluorescence detected after dark adaptation. Actinic light intensity was set to 3,500 μmol·m^−2^·s^−1^ according to Vredenberg (2011). At the end of the experiments roots and shoots were separated and fresh and dry weights determined. Tomato yield was evaluated by measuring the number of fruits and the fresh weight. Quality and antioxidant parameters were also assessed. Total acidity expressed as g L^−1^ of citric acid was obtained by manual titration of tomato juice extract to a pH of 8.2 with 0.1 M NaOH. Ascorbic acid was determined according to Nielsen (2017) and total soluble solids (TSS) by means of a MA871 Refractometer (Milwaukee Instruments, Woburn, MA, USA). Lycopene content was determined according to the reduced volumes of organic solvents described by Fish et al. (2002).

### Data Analysis

Experimental data were tested for normality distribution (Shapiro–Wilk test) and the means compared through analysis of variance (ANOVA). *Post-hoc* test was performed to test the statistical significance (Tukey's HSD test, *P* < 0.05). Principal component analysis (PCA) was used as an exploratory tool to assess the correlation of variables with HA application. XLStat software (Addinsoft) was used for all statistical analyses.

## Results

### ESI FT-ICR MS

FT-ICR MS analysis yielded 9,331 molecular formulae which were assigned with an RMS of 0.17 ppm. The compounds not assigned to a molecular formula represent 10.8% of the total (No hit), highlighted in red in Supplementary Figure 1. The relative abundance weighted average of the molecular weights was 384 m/z with an average of 22 C atoms and 16 equivalent double bonds (DBE) (Supplementary Table 1). The subdivision into groups showed that CHO formulae were the most abundant (47%), followed by CHON (33.6%), CHOS (4.94%), and CHONS (3.72%) (Table 1). The first two groups showed a similar average molecular weight which was lower in CHOS and CHONS where a smaller number of carbon atoms was also observed, thus indicating the presence of smaller molecules in the less represented groups. The low average molecular weight supports the hypothesis of supramolecular aggregation of small molecules dynamically associated through hydrogen bonds, π-π stacking and van der Waals interactions as previously suggested (Piccolo, 2001; Sutton and Sposito, 2005) and reported in heavy oil asphaltenes (Gray et al., 2011; McKenna et al., 2013).

**Table 1:** Group distribution of Idaho HA FT-ICR MS spectrum after molecular formula assignment.

**Group**	***N* peaks**	**% R.A**.	**Avg m/z**	**W. Avg m/z**	**W. Avg C#**	**W. Avg DBE**	**W. Avg H/C**	**AI**
CHO	4,447	47.0	409	369	21	16	0.81	0.58
CHON	3,229	33.6	404	362	20	16	0.66	0.67
CHOS	882	4.94	343	326	17	13	0.76	0.73
CHONS	773	3.72	385	344	17	15	0.99	0.65

*R.A., Relative abundance; Avg, Average; W. Avg, Weighted average; C#, Carbon number; DBE, Double bond equivalent; AI, Aromatic index*.

Up to 50% of the relative abundance (scaled to the 100% peak in each spectrum) corresponded to species assigned to the CHO class having 3–10 oxygen atoms and the CHON class having 4–7 oxygens and 1 nitrogen atom (Table 2). The presence of 15–18 equivalent double bonds suggests the aromatic properties of these molecules. The elemental composition of HA calculated from the FT-ICR data was consistent with the combustion results. S content, however, was partially underestimated.

**Table 2:** Parameters for the main class distribution contributing up to 50% of the relative abundance.

**Class**	***N* peaks**	**% R.A**.	**W. Avg m/z**	**W. Avg C#**	**W. Avg DBE**	**W. Avg H/C**
O6	348	6.32	363	21	16	0.77
O7	339	6.31	382	22	16	0.74
O5	379	6.27	346	21	15	0.80
O4	392	5.24	336	22	16	0.87
O8	297	4.70	398	22	16	0.75
O9	286	3.64	426	23	17	0.75
N1 O5	200	3.49	352	21	16	0.67
N1 O6	189	3.47	370	21	16	0.64
O3	378	3.24	325	22	15	0.99
N1 O4	172	3.01	325	20	15	0.67
N1 O7	183	2.44	393	22	17	0.65
O10	240	2.43	450	23	18	0.74

*R.A., Relative abundance; Avg, Average; W. Avg, Weighted average; C#, Carbon number; DBE, Double bond equivalent*.

**Figure 1:**
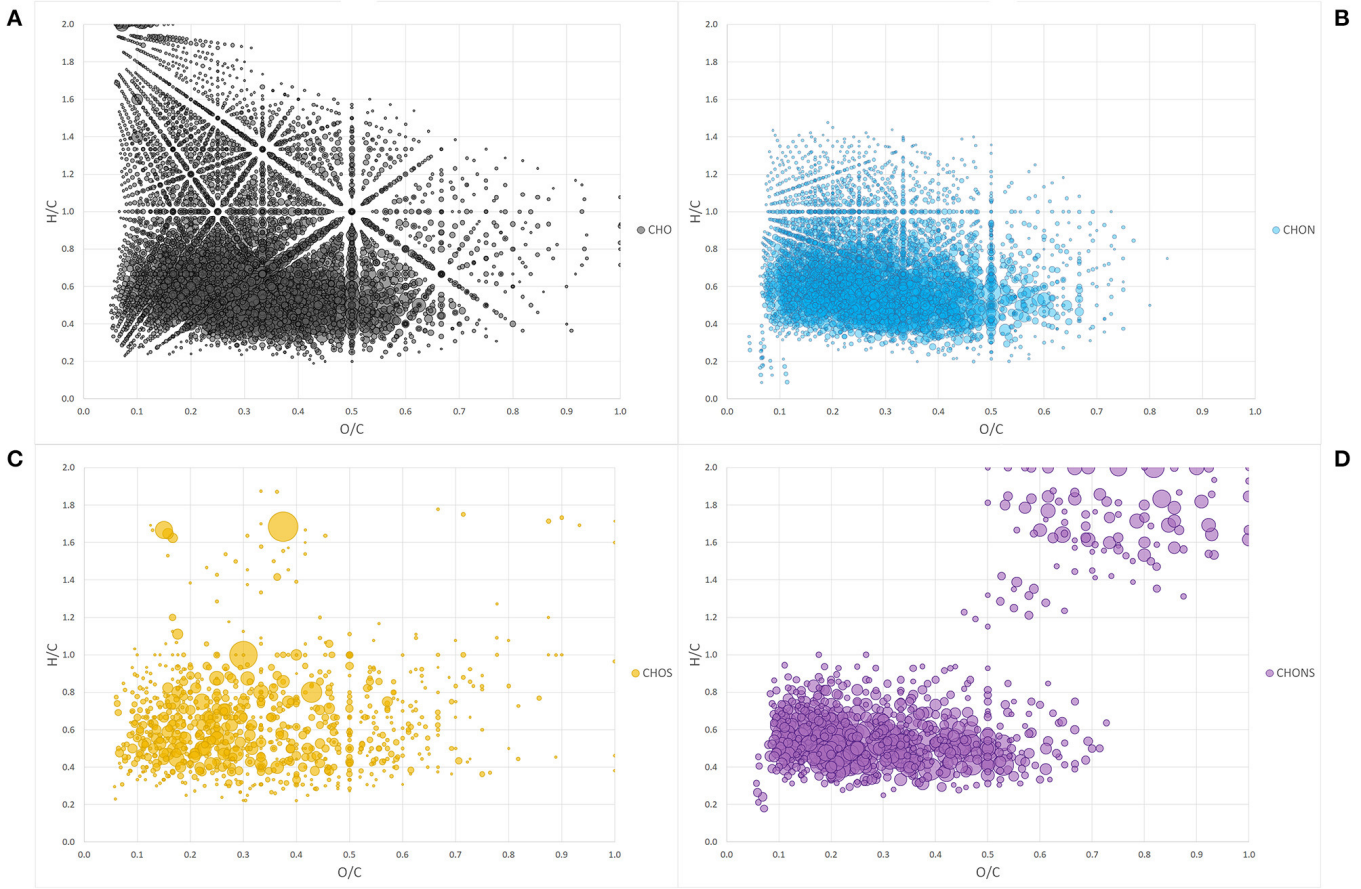
Van Krevelen diagram of heteroatom class groups: CHO **(A)**, CHON **(B)**, CHOS **(C)**, CHONS **(D)**. Each bubble is a molecule assigned and the size of the bubbles is a measure of their relative abundance.

The Van Krevelen diagram containing all the peaks did not allow an immediate visual evaluation as the high number of identified components were superimposed and dispersed along both coordinates (Figure 1A). However, the comparison of single heteroatomic groups revealed that most of the molecules are grouped in proximity of the *x, y* intercepts, extending up to values of 0.65 for O/C and 0.9 for H/C, except in the CHO group (Figures 1B–D).

By assigning the compositional space to areas defined by specific H/C and O/C ratios, it was possible to group the molecules into typical classes of discrete organic molecules such as lignin, lipids, proteins, carbohydrates, condensed aromatic structures (CAS), and unsaturated hydrocarbons (UHC).

**Figure 2:**
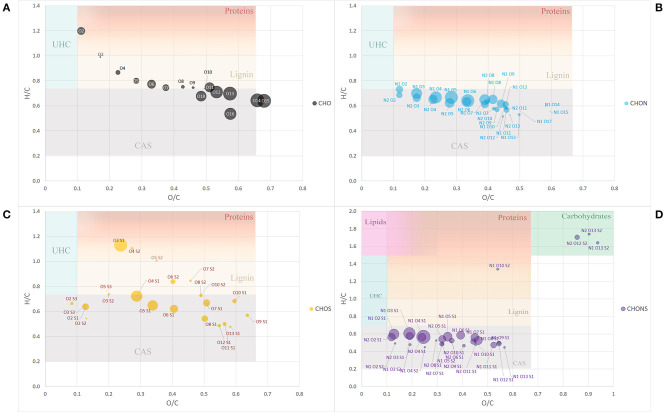
Van Krevelen diagram of class group molecules arranged by the same heteroatoms number. CHO **(A)**, CHON **(B)**, CHOS **(C)**, and CHONS **(D)**. Size of the bubbles is a measure of their relative abundance. Area identified by H/C and O/C ratios and belonging to different type of organic compounds (proteins, lignins, catbohydrates, lipids, CAS, and UHC) have different background color. Fading colors indicate overlapping of different area.

To further simplify the information visualized in the chart, the data reduction of Van Krevelen points was performed by gathering molecules in classes of compounds with the same heteroatomic number (Figure 2). Most of the classes belonging to the group of CHO molecules fell within the lignin compounds, particularly those with a lower number of oxygens ranging from 3 to 10 oxygen atoms, while those with a greater number of O atoms, the most abundant ones, lay in the CAS area (Figure 2A).

The classes belonging to the CHON compounds largely concentrated in the CAS area possessing 1 N atom and oxygen atoms ranging from 3 to 8 (Figure 2B). On the other hand, heteroatomic compounds showing 1 or 2 S atoms were distributed more uniformly among compounds belonging to the CAS category, lignin derivatives and protein-derived structures (Figure 2C).

Finally, the compounds showing the largest heteroatomic distribution appeared in the region belonging to the CAS area with a small number of classes representative of more labile structures such as carbohydrates (Figure 2D).

**Figure 3:**
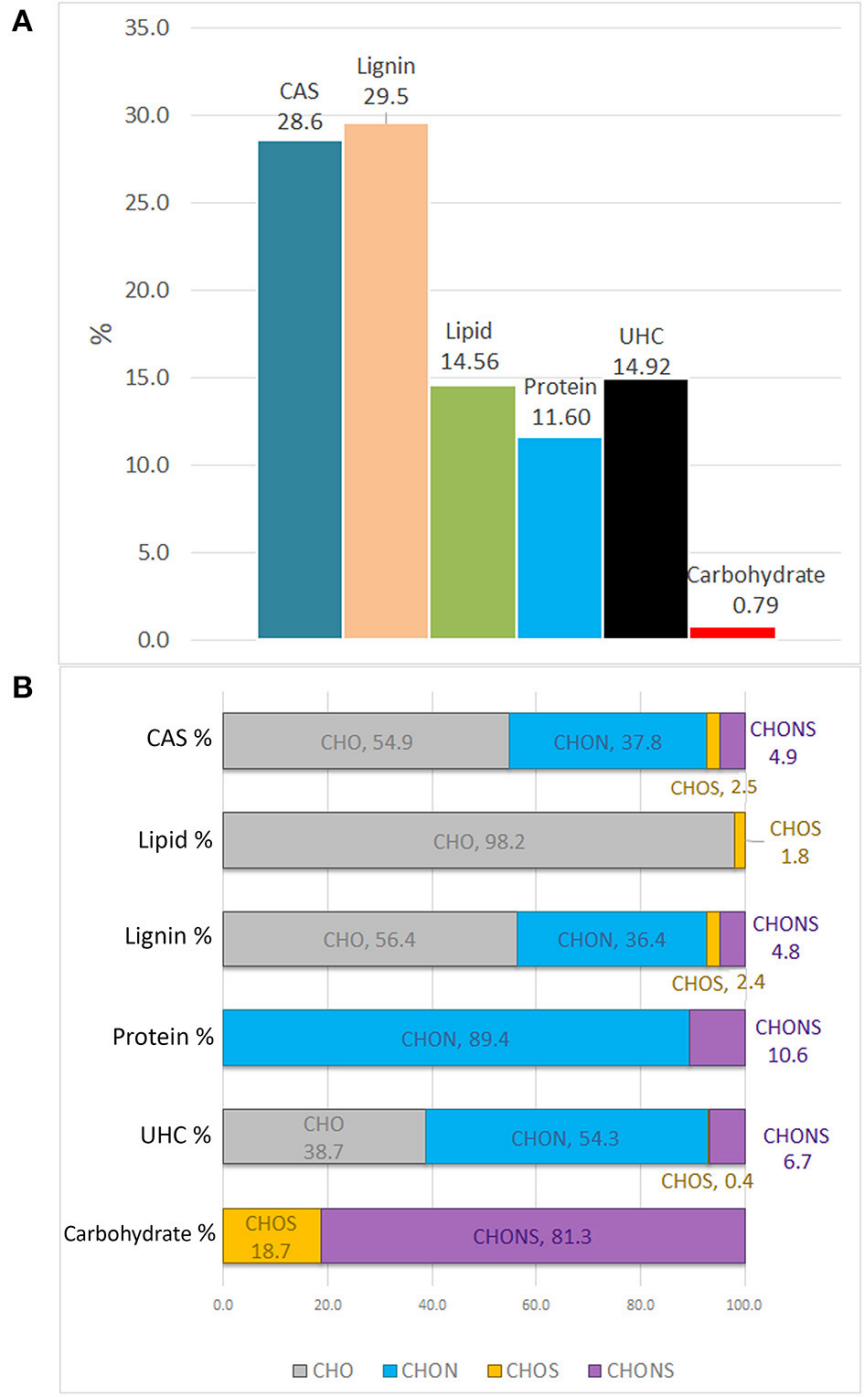
Van Krevelen proportion of different organic compound classes expressed in percentage, as calculated by H/C and O/C ratios **(A)**. Contribution of heteroatoms groups to each compound class **(B)**.

The molecular distribution for each class of compounds calculated by the H/C and O/C ratio are summarized in Figure 3A. The most represented compounds were those falling within the lignin and condensed aromatic structures, quantified at 29.5 and 28.6%, respectively, followed by a smaller proportion in unsaturated hydrocarbons, lipids, proteins, and carbohydrates.

The elemental composition of each biomolecule group is illustrated in Figure 3B. It is interesting to note the contribution of all heteroatomic classes to the CAS, lignin and UHC groups in which CHO structures add up to ~39–55%, whereas the carbohydrate group mainly contained CHONS and CHOS structures but lacked CHO structures. As expected, CHON structures are largely associated with proteins. Lipids were almost exclusively composed of CHO molecules.

By matching FT-ICR molecular formulae by means of online chemical databases, we found that most of the lignin structures corresponding to FT-ICR assignments were represented by flavonoid and isoflavonoid phenolic compounds, some of them falling under bioactive plant and microbial metabolites (Supplementary Table 1). The major contribution came from both the CHO and CHON series, even though N-containing compounds belonged to more differentiated classes such as alkaloids, benzamides, and several nitrobenzene analogues in which the polar character of nitro groups confers a strong electron-withdrawing capacity and reactivity. In general, they can be classified as aromatic amines because of their relevant hydrogen-deficiency. Polyphenol-peptide reactions that produce condensed structures are likely to occur naturally. The CHON series occurred primarily in the area delimiting CAS and lignin structures (Figure 2). On the other hand, the CAS group contained mainly CHO and CHON formulae. Part of the nitrogen associated with HS is expected to be released when HA is separated from fulvic acid by acidic hydrolysis of peptide bonds. However, for amino acids directly bonded to phenolic rings, N may still exist as an acid-insoluble complex, as confirmed by the infrared spectra in the typical absorption of the peptide bond at 1,417 cm^−1^ (Figure 6), or as part of a heterocyclic ring as a stable –NH such as in indole (Stevenson, 1994). Condensed aromatic structures were the second most abundant group identified in the FT-ICR data and showed a substantial presence of quinone-derived structures along with alkaloids, flavonoids, and PAH, most of them identified as potentially bioactive compounds of plant, fungal, and bacterial origin (Senthamarai et al., 2003). Interestingly, some of them derive from a marine and freshwater environment and show antimicrobial and antioxidant activity (Namikoshi, 2006; Sturdy et al., 2010). Similarly, the UHC group also contained an abundance of quinone-derived structures where the presence of nitrogen gives pyridine aromatic analogues.

The most abundant structures identified in the lipid group were saturated fatty acids derived from fossilized plant waxes, hydroxy acids, and dicarboxylic acids. It is worth mentioning the large abundance in this list of polyunsaturated arachidonic acid whose inclination to react with molecular oxygen suggests its contribution to oxidative stress through the effect on H^+^ channel activity (Henderson et al., 1997).

Protein class contributions were identified mainly in alkaloids, indoles, heterocyclic amines, and amino compounds and possibly bioactive quinoline derived structures, while the carbohydrate group was the less represented in FT-ICR and the most challenging to assign. However, even if small, this group was dominated by CHONS structures, probably sugar sulfates, sulfonates, thiocarbonates, or isothiocyanate derivatives such as glucosinolates, but the lack of specific structural analyses, makes it difficult to draw conclusions about the identity of these compounds.

### ^13^C CPMAS NMR

**Figure 4:**
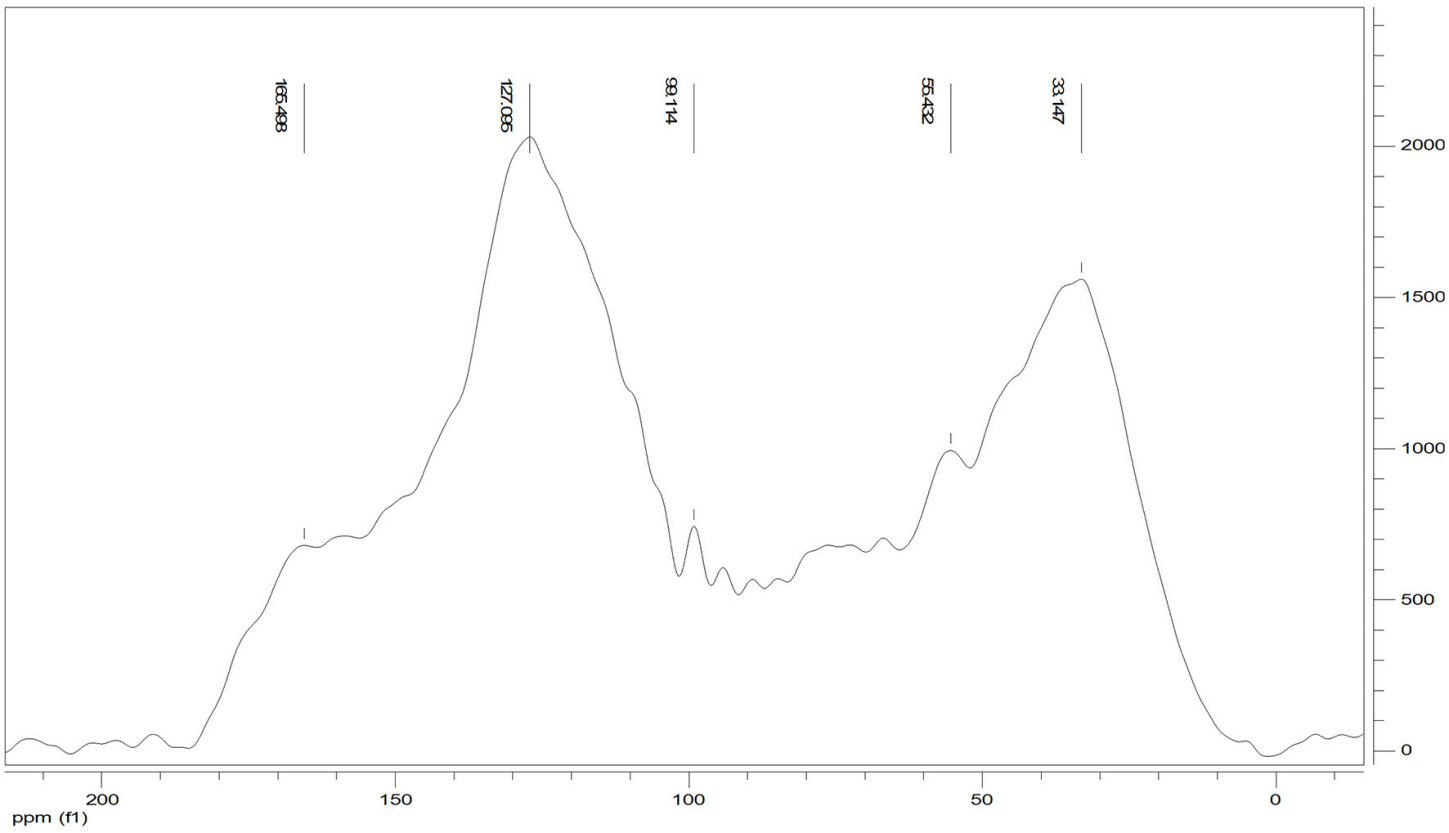
^13^C CPMAS NMR spectrum of Idaho HA.

The ^13^C CPMAS NMR of the Idaho humic acid is shown in Figure 4. Two broad resonances appear to be predominant, the first in the range 10–45 ppm where the highest peak at 33 ppm was indicative of methyl groups belonging to alkylic structures such as lipidic compounds, and the second in the range of 110–145 ppm where the highest signal appearing at 127 ppm indicated the abundance of protonated aromatic rings.

The relative carbon distribution over the chemical shift is summarized in Table 3. The functional groups most represented were the Alkyl-C and Aryl-C, whose regions accounted for 23.6 and 31% of the total area, respectively, followed by the O-Alkyl-C (12.2%), the O-Aryl-C (9.8%), and Carbonyl-C (8.8%) as the most abundant groups. The lower resonances in the O-Alkyl regions, assigned to mono- and polysaccharidic structures mainly derived from plant cellulose, are not resolved in any predominant peak. However, their presence was indicated by the peak at 99 ppm assigned to the anomeric carbons. Methoxyl-C in the range 45–60 ppm, which accounted for 8% of the total area, confirmed the presence of lignin material as the peak at 55 ppm was associated with methoxyl groups substituted on the aromatic core as well as the side chain of lignin monomers. Phenolic compounds added up to lignin aromatic rings as O-substituted C, however this region was mostly overlapped by the Aryl-C region.

**Table 3:** Relative contribution (%) of main C structures over chemical shift regions (ppm) calculated from ^13^C CPMAS NMR of Idaho HA sample and structural indices derived from spectral areas.

	**Carbonyl-C**	**O-Aryl-C**	**Aryl-C**	**O2-Alkyl-C**	**O-Alkyl-C**	**Methoxyl-C**	**Alkyl-C**				
**Sample**	**210–165**	**165–145**	**145–110**	**110–95**	**95–60**	**60–45**	**45–10**	**HB**	**AlR**	**LigR**	**AI**
IDHA	8.8	9.8	31.0	6.7	12.2	8.0	23.6	1.8	1.25	0.81	0.5

*HB, Hydrophobicity index; AlR, Alkylic ratio; LigR, Lignin ratio; AI, Aromaticity index*.

Carbonyl-C was visible as a shoulder in the range 165–210 ppm and the peak at 165 ppm was indicative of carboxylated functions in aliphatic chains as well as in protein derived compounds.

Structural indices calculated from the ^13^C spectrum indicated the mainly aromatic character of the HA where the LigR suggested that preservation of lignin structures happened through the advanced oxidative transformation and stabilization degree of this material. In addition, the AlR and HB indices highlighted the contribution of aliphatic and olefinic structures to the hydrophobicity degree (Table 3).

### Molecular Mixing Model: MS and NMR Data Comparison

#### Elemental Composition

The bulk elemental composition comparison suggested that the FT-ICR values were in good agreement with the elemental analysis in relation to the capacity of this instrument to delve deeper into carbon chemistry (Table 4). Discrepancies arose when heteroatoms were considered as the N and S content appeared to be underestimated in the MS data. However, the conservative approach used in the ESI MS analysis was intended to preserve the quality and robustness of the results while keeping the error as low as possible. This was consequently reflected in the molar ratios calculated by each technique and suggested a H– deficiency in the MS data, possibly due to the exclusion of hydrocarbons with only C– and H– from the analysis, which are generally not considered as constituents of organic matter. O/C and N/C ratios were overall in agreement across the analyses as well as the aromaticity index that resulted in a slightly higher value when the FT-ICR result was compared to the NMR mixed model value.

**Table 4:** Elemental composition of Idaho HA as determined by elemental analysis (EA), FT-ICR ESI MS, ^13^C CPMAS NMR (through molecular mixing model MMM).

	**C%**	**H%**	**O%**	**N%**	**S%**	**H/C**	**O/C**	**N/C**	**S/C**	**AI**
EA	50.1	–	–	2.03	1.59	–	–	0.04	0.032	–
FT-ICR	50.2	32.9	15.2	1.38	0.29	0.65	0.30	0.03	0.006	0.66
^13^C NMR MMM	–	–	–	–	–	1.18	0.34	0.08	–	0.5

#### Molecular Distribution

**Figure 5:**
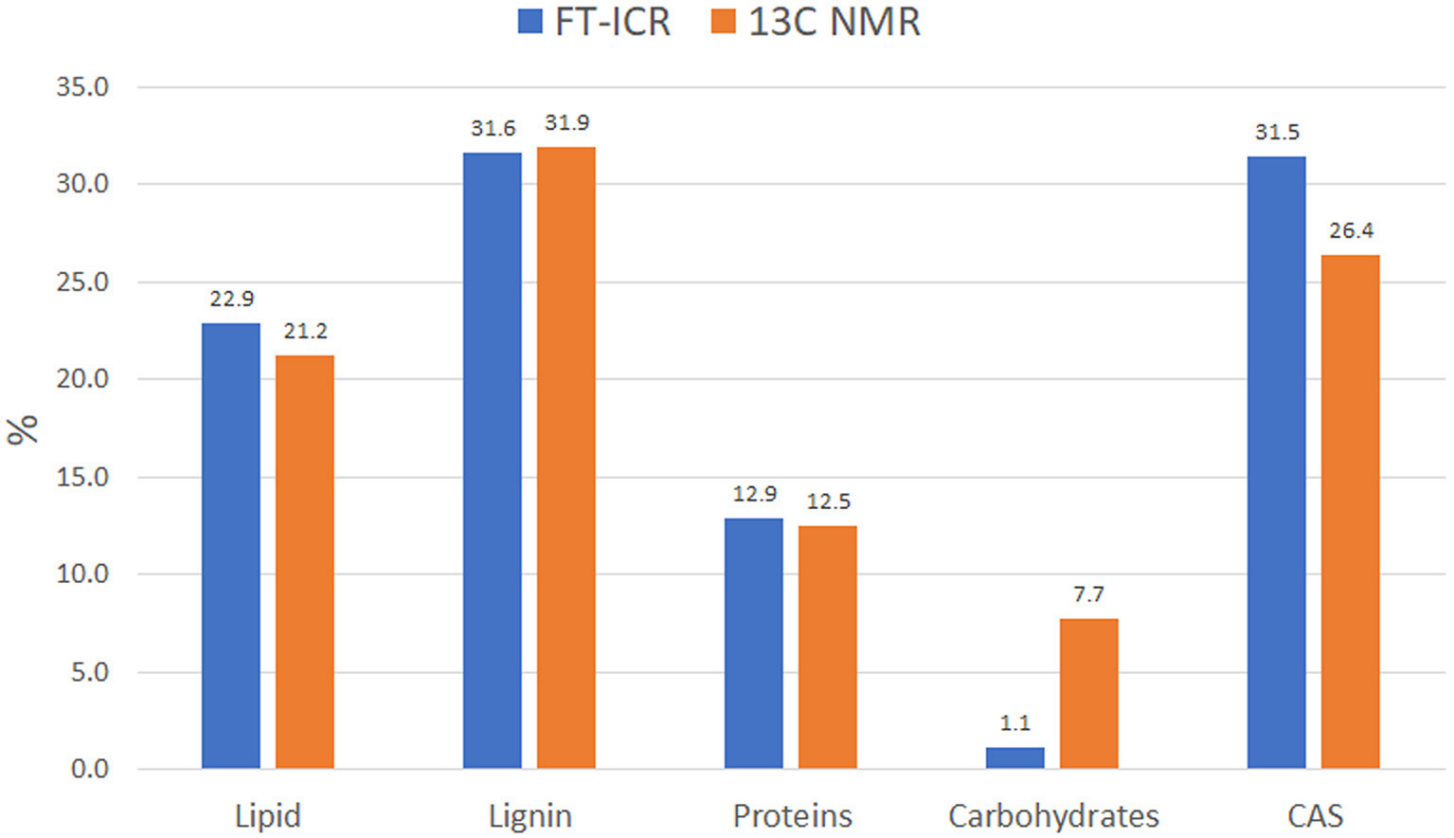
Idaho HA molecular distribution into the main biomolecule structures as observed by FT-ICR and ^13^C NMR. Data computed through MMM for both analyses.

Data reduction and aggregation along with the molecular mixing model approach allowed the comparison of NMR and FT-ICR data in terms of individual bio-molecule structures. To make the comparison matching accordingly, the NMR carbonyl value was added to the aliphatic value, while the MS UHC value was combined with the lipid group. Finally, the Char classification in the NMR is referred to as CAS in the MS analysis. There was a close agreement of NMR and MS biomolecule groups, except for the carbohydrates class, which was underrated in the FT-ICR results (Figure 5). This trend has been observed previously by other authors and seems to be ascribed to ionization efficiency of this biochemical class (Hockaday et al., 2009). The difference observed in the CAS value between the two techniques can be ascribed to a minor efficiency of cross-polarization technique to correctly represent carbons that are not closely associated with protons, like those of condensed aromatic rings. Consequently, aliphatic carbons could have been preferentially cross-polarized over condensed aromatics and that could have resulted in the underestimation (Kramer et al., 2004).

### FT-ATR

**Figure 6:**
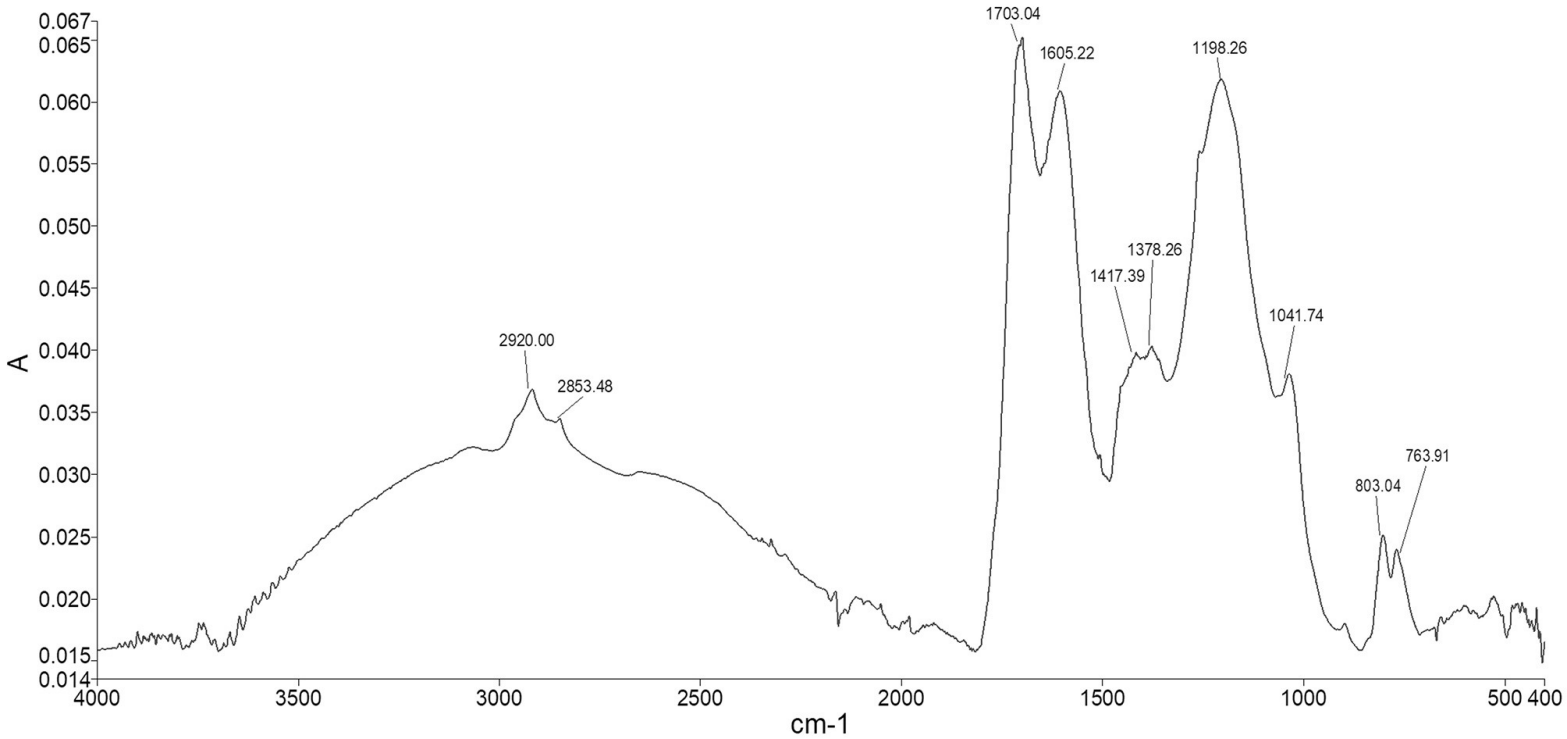
Idaho HA FT-ATR spectrum.

The spectrum of Idaho HA (Figure 6) showed the typical adsorption bands of humic material. The two peaks appearing at 2,920 and 2,853 cm^−1^ were assigned to both the symmetrical and asymmetrical stretching vibrations of methyl and methylene functions of aliphatic structures, including fatty acids, waxes, higher alkanes and other naturally occurring polyesters. The broad shoulder ranging from 2,500 to 3,500 cm^−1^ represented hydroxyl groups belonging to alcohols, phenols, and carboxylic acids. The fingerprint region showed two typical adsorptions at 1,703 and 1,605 cm^−1^ where the stretching of C=O and the vibrations of carbonyl occur, indicating the presence of carboxylic acids, further confirmed by the weak peak at 1,417 cm^−1^, which can be alternatively attributed to amide II bond stretch. The quite intense peak at 1,198 cm^−1^ and the shoulder peak appearing at 1,041 cm^−1^ were assigned respectively to phenolic –OH of lignin structures and the C–O stretching of polysaccharidic compounds, such as cellulose and hemicellulose derivatives. The two peaks in the lower fingerprint region at around 803 and 763 cm^−1^ were assigned to –CH vibrations of substituted benzene rings belonging to both aromatic and phenolic derivatives.

### Plant Growth Bioassay

To test the biological activity of the HA extract, plant morphological traits were investigated. The final goal of the application of HA is the positive economic impact on production. Therefore, yield and fruit quality parameters were also evaluated.

Plants grown under HA treatment showed the best general performances, especially when NPK nutrient supply was reduced to half or a quarter in combination with HA application. The differences observed in shoot biomass showed that the largest value was produced by the 50 HA treatment, followed by 25 HA treatment (Table 5). Root biomass was generally positively affected by the application of HA. Root weight, in particular the dry weight, was significantly affected by the presence of HA. The best overall performance was displayed by the 50 HA treatment. Even though the HA supplied under full nutrition resulted in a detrimental effect for several parameters observed, root growth was still positively influenced by the addition of the extract. Chlorophyll content showed an increasing trend when HA was supplied at increasing nutritional doses, however, the differences were not statistically significant. A decrease in chlorophyll fluorescence is an indicator of an ongoing physiological stress. As expected, the control treatment under full nutrition showed the least stress condition, followed by 25 HA and 50 HA that showed the ability to better cope with the stress when compared to the relative controls. Conversely, the drastic decrease in the fluorescence signal measured for 100 HA treatment indicated a reduced photosynthetic efficiency (Table 5).

**Table 5:** Plant morphological mean data, tomatoes yield, and quality parameters.

	**H**	**CHL**	**CHL FL**	**SHOOT FW**	**SHOOT DW**	**ROOT FW**	**ROOT DW**	**TOM N**	**TOM FW**	**TA**	**TSS**	**AsA**	**Lycopene**
**Treatment**	**cm**	**μmol m^**2**^**	**Fv/F0**	**g**	**g**	**g**	**g**		**g**	**g/L citric a**.	**Brix**	**mg/g FW**	**mg/100 g FW**
25	6.85	401.9	2.95	7.76	1.73	4.45	0.51	13	3.25	4.0	5.5	0.46	11.5^*^
50	7.3	403.8	2.94	8.04	1.69	4.21	0.78	16	5.06	4.0	6.2	0.48	10.0
100	7.36	423.0	3.02^*^	9.10^*^	1.88	5.14	0.66	32	5.65	4.1	5.4	0.52^*^	11.8
25 HA	7.39	425.3	3.00^*^	9.93^*^	2.07^*^	3.94	0.79^*^	16	4.29^*^	3.9	6.2^*^	0.49^*^	10.6
50 HA	7.84	428.1	2.96	10.93^*^	2.12^*^	5.09^*^	0.91^*^	19	5.22	5.5	6.0	0.53^*^	12.3^*^
100 HA	7.02	399.3	2.73	7.83	1.68	4.57	0.81^*^	28	6.29	5.2	7.1^*^	0.47	12.3^*^

*H, height; CHL, Chlorophyll; CHL FL, Chlorophyll fluorescence; FW/DW, Fresh/Dry weight; TOM N, Tomatoes number; TA, Total acidity; TSS, Total soluble solids; AsA, Ascorbic acid. Significant difference at P < 0.05 (Tukey's test) are indicated by asterisk*.

Tomato production and quality assessment results are summarized in Table 5. Yield obtained by HA-treated plants showed an increased number of tomatoes produced, up to +19 and +16% in 25 HA and 50 HA treatments, respectively. The trend was not continued when full nutrition was supplied, in which the application of HA decreased the numbers of tomatoes by 13% when compared to the relative control. However, tomato fresh weight was increased in all HA treatments, up to a +24% in the 25 HA treatment.

The fruit quality assessment involved the analysis of acidity, the total soluble solid content (i.e., Brix) and the antioxidant activity measured by lycopene and ascorbic acid production (Table 5). Total acidity increased as a result of the application of HA at half and full nutritional strength, while the soluble solid content increased in the 25 HA and 100 HA treatments, +12 and +24%, respectively, as compared to non-treated plants. Lycopene content increased significantly only in the 50 HA and 100 HA treatments. Except for the full nutritional level (100 HA) where there was a significant decrease, the application of HA increased the ascorbic acid concentration up to 10% in the 50 HA treatment as compared to respective control.

**Figure 7:**
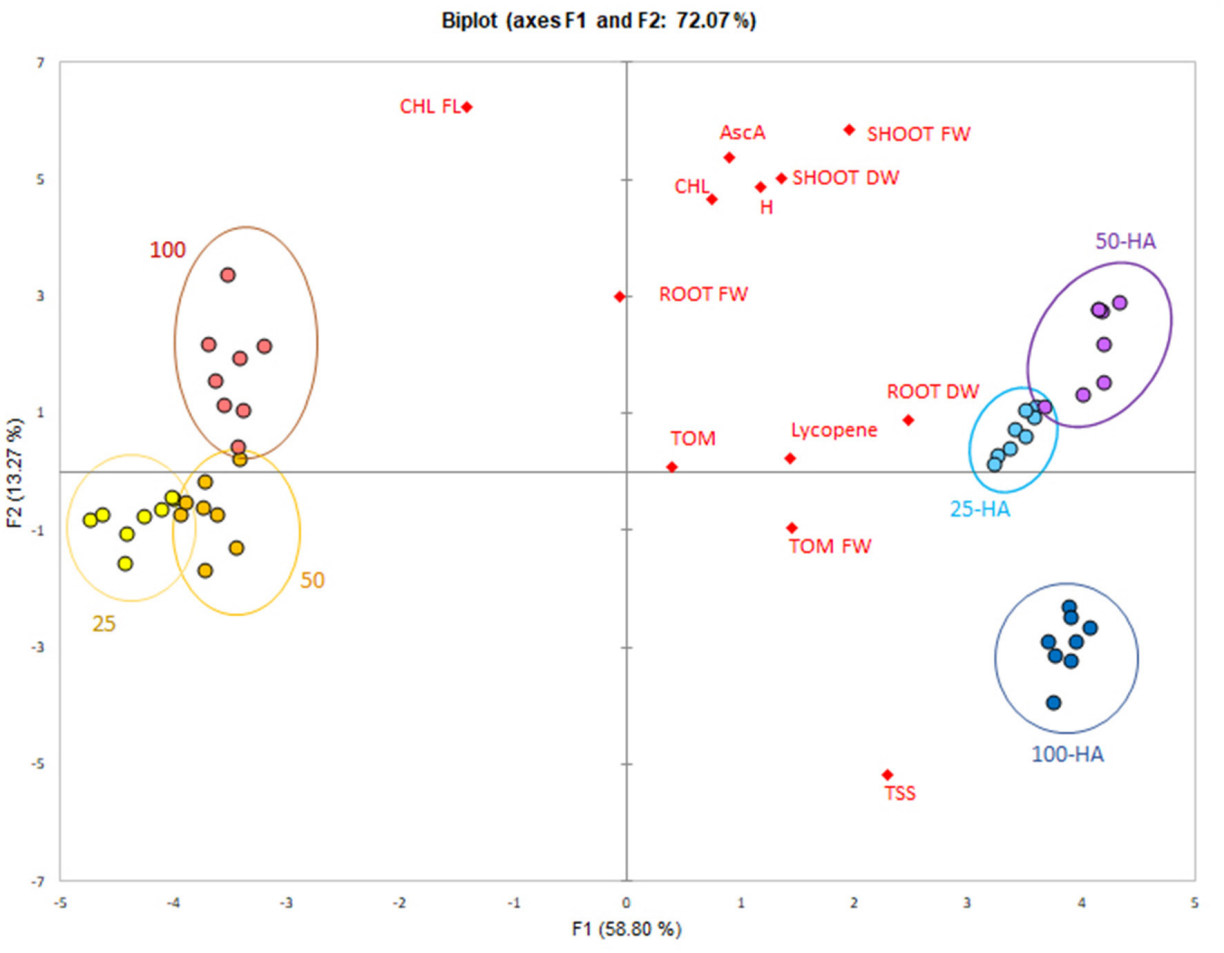
PCA score-plot of plant bioassay. Measured parameters correlation with the application of humic acids at different nutritional levels.

The data reduction through principal component analysis allowed the determination of the variables most influenced by the HA application. Humic treatments were clearly separated from the controls along the first principal component that explained 58.8% of variability (Figure 7). All the variables were spread mostly along the second principal component and showed a positive correlation with 25 HA and 50 HA treatments. Root dry weight was the strongest correlating parameter, while chlorophyll, root fresh weight and ascorbic acid appeared less important for the purpose of biostimulation.

## Discussion

Optimization of nutrient use efficiency represents an important strategy to reduce the environmental cost generated by harmful contamination of groundwater and atmosphere that mineral fertilizers produce when used in excess to maximize crop production (Conley et al., 2009). The use of HS as biostimulant represents a cost-effective and environmental-friendly tool to improve nutrient uptake by promoting sustainable agricultural practices. Indeed, HS affect nutrient complexation and act as natural chelates (Garcia-Mina et al., 2004; Tomasi et al., 2014) but they also induce plant metabolism changes. HS stimulate active proton extrusion from the root plasma membrane by the activity of H^+^ –ATPase resulting in the generation of a transmembrane potential involved in the cell elongation and active uptake of nutrients (Varanini et al., 1993; Canellas et al., 2002; Zandonadi et al., 2007; Jannin et al., 2012). More recently it has been observed that HS performances increase when a stress condition is present. Jindo et al. (2016) demonstrated that application of HS in the presence of low phosphorus availability induces high-affinity P_i_ transporters in plant roots thereby enhancing P uptake. Tavares et al. (2019) found that HA stimulated ${\text{NO}}_{3}^{-}$ uptake after 96 h of N deprivation. Additionally, the chemical nature of HS that differs for each source plays a key-role and often leads to practical ineffective results. In this SAR study the biostimulant activity of an ore-extracted and purified HA was evaluated in a tomato pot experiment and the HA chemical proprieties were characterized by means of high-resolution MS, ^13^C NMR, and FT-IR. The chemical nature of HA was further analyzed for the recognition of specific biochemical structures potentially involved in tomato plant morphological and productivity responses, including an increase in defense mechanism parameters.

The application of HA confirmed its ability to stimulate tomato plant growth. HA treated plants yielded more tomatoes than control plants when combined with lower nutritional dose. Comparison of the control under 50% of nutritional dose with the HA treatment at 25% of nutrition supplied showed a similar production rate and similar photosynthetic activity, indicating the ability of HA to alleviate the stress condition and to partially reduce the amount of fertilizer required to obtain comparable results.

Tomato fruit quality improvements were also reported in all HA treatments. Although the application of HA was less effective under full nutrition in terms of some morphological parameters in this treatment, HA may have caused the plants to shift to an energy conservation strategy that entailed less vegetative growth but promoted mainly fruit development. In fact, even if tomatoes produced in HA100 were slightly less in number than control, their fresh weights were larger by 10%. This conclusion was also supported by the content of total soluble solids, lycopene, and total acidity that in 100 HA tomatoes outperformed the respective control.

The application of HA overall increased the content of measured antioxidants as a result of plant defense system activation. Ascorbic acid, which as the primary plant antioxidant contributes to the reduction of the oxidative damage, may have allowed a stronger response of HA treated plants to the nutrient stress condition. When compared to relative controls, the HA treatments showed a significant lycopene increase only at higher nutritional input. The increased stress due to deficient nutrition is supposed to increase the antioxidant content at lower NPK supply, but conversely to what expected, lycopene increased as nutritional dose increases, in both controls and HA treatments. Our results are in accordance with Koleška et al. (2017). The higher K and P fertilization could be potentially responsible for the described trend because they have been reported to positively influence lycopene content in tomato as their supply is increased (Zdravković et al., 2004; Ramírez et al., 2009).

The FT-ICR MS analysis of the HA helped provide an understanding of the composition and distribution of major biomolecule classes present. This ore-extracted HA consisted of more than 10,000 small molecules with an average m/z of 384 and are representative of plant and microbial aromatic biomolecule derivatives. Together with a marked presence of aliphatic compounds, these components confer on this HA a distinctive aromatic and hydrophobic character. The ^13^C CPMAS NMR supported this outcome showing that relative carbon distribution is concentrated mostly in the aliphatic and aromatic region where the presence of peaks at 56, 127, and 165 ppm have been previously associated with lateral root stimulation bioactivity (Aguiar et al., 2013). On the other hand, it highlighted the preservation of a modest carbohydrate component that was not found in the FT-ICR MS. The molecular mixing model was used to predict the molecular allocation of biomolecules by applying FT-ICR MS data to the model and comparing the results to the NMR data. The model showed a close match in the distribution of biomolecules, except for carbohydrates, confirming the preferential ionization, in the ICR cell, of some classes of compounds such as CAS and lipids. Nonetheless, the correspondence in all the other groups overall proved the validity of the model comparison.

Online database searches of the molecular formulae identified several bioactive molecules belonging to the lignin derived flavonoids class, quinone-derived structures, and other molecules belonging to CAS and lipids that are potentially involved in the oxidative stress modulation.

Flavonoids are a big family of phytochemicals involved in the plant defense mechanism while coping with a stress condition (Cetinkaya et al., 2017; Trejo-Téllez et al., 2019). They are synthesized through the phenylpropanoid pathway and exhibit ROS scavenging properties assisting plants in tolerating and escaping external biotic and abiotic stresses (Treml and Šmejkal, 2016). As their role as antioxidants is widely recognized (Pietta, 2000), their occurrence in this humic extract supports the biostimulant action that HA exert on plant fitness. However, they can also be involved in priming plant stress machinery as described by Canellas et al. (2020).

Several authors described the prooxidant and antioxidant properties of phenols, alkaloids, and quinones (Azam et al., 2003; Pietsch et al., 2011; Kurutas, 2015). Polyphenols are considered antioxidants, but they not only might undergo oxidative reactions, but when applied externally, their allelochemical biological role and negative impact on target organisms should be considered. These includes impacts such as ROS generation, inhibition of cell division and reduced photosynthetic rates, among others. Secondary metabolites and particularly phenols can often demonstrate prooxidant activity by releasing a hydrogen atom and producing a reactive semiquinone radical capable of reducing oxygen to ^·^${\text{O}}_{2}^{-}$ which can be further converted to other detrimental ROS including H_2_O_2_ and ^**·**^OH^−^ by scavenging of other phenols (Grace, 2005; Gniazdowska et al., 2015).

Quinones on the other hand can act as prooxidant and have been proposed as potentially responsible for triggering the ROS production in plant, by acting as electron shuttles due to their oxidizing/reducing capabilities (Lamar, 2010; Lv et al., 2018). However, the antioxidant function of isoprenoid quinones has been recently described by Kruk et al. (2016). They have, indeed, an important biological role as redox co-enzymes and vitamin constituents. Zhang et al. (2018) demonstrated that HA contained redox-active groups and exhibited redox potentials between −0.36 and −0.28 V suggesting their role as redox mediator in enhancing multiple microbial reductions, thereby affecting various biogeochemical processes. Nonetheless, Zykova et al. (2018) attributed the radical scavenging property of several HA to the presence of condensed aromatic structures such as semi-quinone type and phenoxyl type radicals.

The presence in HA of molecules that can act transiently as antioxidant or prooxidant depending on the environmental constraints, could explain, at least in part, the bioactivity effect through the modulation of ROS accumulation in plant. Despite the damage that ROS exposure might have in the oxidative process, ROS have an important role as signaling molecules, often leading to the conferment of tolerance to environmental stresses (Balasubramaniyam, 2015). Whether the exposure to stress promotes toxicity or acclimation strategy depends on the homeostasis balance between ROS production and ROS scavenging that eventually produces a shift in the regulatory role of ROS from cell signaling to the negative physiological effects (García et al., 2016a).

HS have been found to increase the ROS levels by acting as a mild stressor by triggering the plant defense system. García et al. (2012) described the increase of ROS in rice roots as a consequence of HA application. Similarly, Mehrasbi et al. (2018) found that HA affected ROS production in algae. However, the pre-teatment with HA has been found to mitigate the presence of major abiotic stresses induced by PEG (García et al., 2016b) as well as salinity, drought and heavy metals (Canellas et al., 2020), resulting in higher transcription level of genes involved in stress perception.

All the molecules found in this study could be involved in both the determination of a eustress, where the final effect is somehow beneficial for the plant, or in the establishment of a distress, leading to detrimental and irreversible tissue damage (Vargas-Hernandez et al., 2017).

In our study, plants under nutritional stress performed better when HA was supplied, while plants at full nutrition were not showing a clear advantage from HA application, which might have behaved as a stressor when no other stress was present. On the other hand, all HA treatments showed faster adaptation to the stress condition, particularly when nutrient deficiency occurred. The resulting increased nutrient accumulation and growth of tomato seedlings by application of humic under limited nutrient availability solution was reported by David et al. (1994) and supports our observations. Indeed, the increased root biomass observed was indicative of a better nutrient uptake efficiency and could have resulted from ROS sensitive signaling response to nutrient deprivation that leads to cell-wall relaxation and root growth (Schachtman and Shin, 2007), a process strictly correlated to the activation of plasma membrane H^+^ ATPase reported by several authors (Nardi et al., 2017; Tavares et al., 2017). This hypothesis is in accordance with Cordeiro et al. (2011) that found ROS level increased in the maize root apex upon HA application and a higher transcription of catalase antioxidant enzyme when nitrogen supply was low.

Because the final effect of HS is not solely the consequence of the presence of a single molecule but relies on the complex mix of constituents, how the final effect on plant is modulated is still difficult to predict, but it is likely to be associated with the emerging properties defined by the interaction with plant defense system and the biochemical environment. In fact, lignin, and CAS derived molecules can participate in electron transfer reactions either as donors or acceptors, depending on the presence or absence of specific functional groups. For example, electron transfer mediated by one phenolic hydroxyl group can lead to an oxidized radical, while the presence of two hydroxyl groups on catechol can reduce H_2_O_2_ to H_2_O. However, if ROS are not present, the catechol can reduce molecular oxygen to H_2_O_2_, while the presence of ROS drives the process toward the scavenging reactions as long as homeostasis restoration is achieved (Hadacek et al., 2011). The role of HS electron accepting capacity has been investigated by Yang et al. (2016) who found that quinone moieties were responsible for the high reducing ability of low molecular weight HA such as the ones described in our study. Lv et al. (2018) demonstrated that polyphenol-like compounds with medium oxygen content were the major compounds acting as electron donors in HS. Furthermore, polyphenols such as flavonoids can be involved in nutrient uptake as they form stable complexes with Fe and Al present in insoluble Fe- and Al-phosphates thereby increasing the P solubility for plant uptake (Cesco et al., 2010). Nonetheless, they can also prevent microbial degradation of extracellular phosphatases and organic acids released by roots as a response to nutritional deficiencies (Neumann and Römheld, 2007).

Based on our results, here, we suggest that the balance of flavoinoids and quinones found in the humic extract could have positively modulated ROS signaling involved in plant nutrient uptake and therefore triggered the biostimulant effect observed. While the understanding of mode of action will require further investigation, plant pre-conditioning with HS might represent an important determinant in the adaptive plant defense response and an effective strategy to improve nutrients management and plant productivity.

## Conclusion

The outcomes of this study highlighted the role of HA in enhancing nutrient efficiency uptake. The application of HA at low NPK supply improved tomato yield and plant ability to cope with nutritional stress. Chemical composition revealed the presence of both antioxidants and prooxidant molecules such as flavonoids and quinones and suggested their role as modulators of ROS level in plant by priming plant defense systems and resulting in increased root exploration and antioxidant production. Our results proved that use of HA ultimately leads to a fast and effective response to nutrient deficiency based on increases in plant morphology and productivity.

The implementation of *in silico* technologies represents a valid tool and a promising strategy where combinations of ultra-high mass resolution and complementary techniques will allow a more extensive understanding of molecular composition of HS from different source environments.

## Data Availability Statement

The original contributions presented in the study are included in the article/Supplementary Material, further inquiries can be directed to the corresponding author/s. FT-ICR MS data is publicly-available on the Open Science Framework through DOI: 10.17605/OSF.IO/TF7QB.

## Author Contributions

HM and RL conceptualized and designed the study. HM performed the experimental work, the plant experiment, the analytical data analysis, and statistical tests. AM supported the FT-ICRMS data interpretation. RF did the elemental analysis. HM wrote the manuscript. RL and AM supervised the article. All authors approved the submitted version.

## Conflict of Interest

HM, RL, and RF were employed by the company Bio Huma Netics, Inc. The remaining author declares that the research was conducted in the absence of any commercial or financial relationships that could be construed as a potential conflict of interest.

## Supplementary Material

Supplementary Figure 1 Idaho HA FT-ICR spectrum. “No hit” highlighted in red **(A)**. Distribution of molecular weights vs. intensity for the different heteroatom groups **(B)**.
