# Supplementary figures and images for: Bioactivity of Humic Acids Extracted From Shale Ore: Molecular Characterization and Structure-Activity Relationship With Tomato Plant Yield Under Nutritional Stress
Source: Front Plant Sci. 2021 May 26;12:660224. doi: 10.3389/fpls.2021.660224 (PMC8195337; doi:10.3389/fpls.2021.660224)

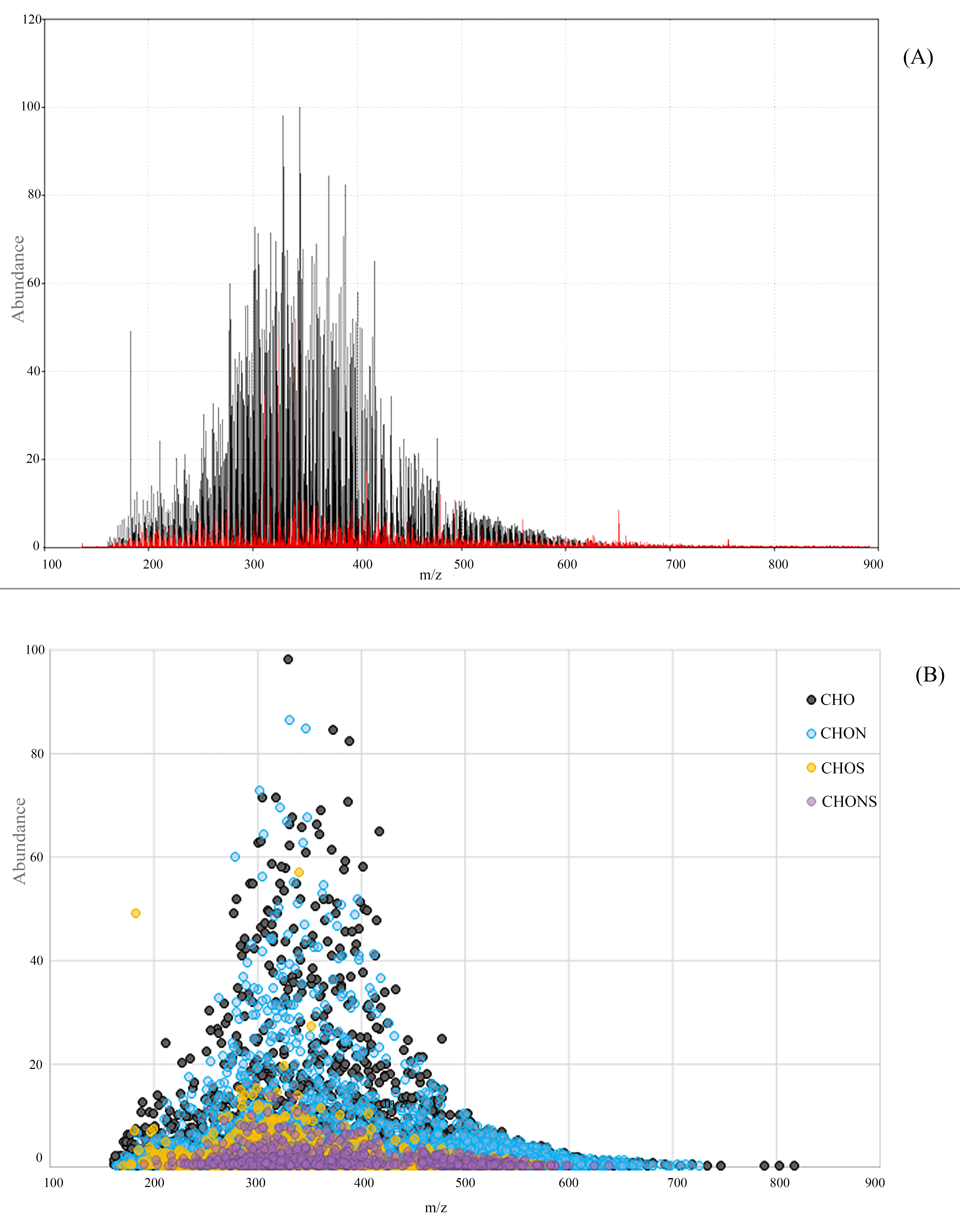

Supplement: Supplementary Figure 1 — Idaho HA FT-ICR spectrum. “No hit” highlighted in red (A). Distribution of molecular weights vs. intensity for the different heteroatom groups (B). [file Image_1.jpg]
