# Supplementary material for: Bioactivity of Humic Acids Extracted From Shale Ore: Molecular Characterization and Structure-Activity Relationship With Tomato Plant Yield Under Nutritional Stress
Source: Front Plant Sci. 2021 May 26;12:660224. doi: 10.3389/fpls.2021.660224 (PMC8195337; doi:10.3389/fpls.2021.660224)
Supplement: Supplementary file 2 [file Table_1.docx]

**Table S1.** FT-ICR MS general information obtained from the total number of peaks assigned.

|  | N peaks | % R.A. | % No Hit | Error (ppm) | W. Avg | W. Avg C# | W. Avg DBE | Aromaticity index (AI) |
| --- | --- | --- | --- | --- | --- | --- | --- | --- |
| Total | 13647 | 100 | 10.8 | 0.17 | 384 | 22 | 16 | 0.66 |
