# Supplementary material for: Bioactivity of Humic Acids Extracted From Shale Ore: Molecular Characterization and Structure-Activity Relationship With Tomato Plant Yield Under Nutritional Stress
Source: Front Plant Sci. 2021 May 26;12:660224. doi: 10.3389/fpls.2021.660224 (PMC8195337; doi:10.3389/fpls.2021.660224)
Supplement: Supplementary file 3 [file Table_2.docx]

**Table S2**. Online database matching of the most abundant molecular formulae for each biomolecule group carried out from FT-ICR MS data.

|  |  |  |  |  |  |
| --- | --- | --- | --- | --- | --- |
| **Lignin** |  |  |  |  |  |
| Formula | compound |  |  |  |  |
| C17H12O6 | 5-(3-phenylprop-2-enoyloxy)benzene-1,3-dicarboxylic acid Fujikinetin | | | | |
| C20H14O5 | diphenyl 2-hydroxybenzene-1,3-dicarboxylate Sophoracoumestan A | | | | |
| C17H12O5 | phenyl 7-methoxy-2-oxochromene-3-carboxylate Haloquinone | | | | |
| C20H14O4 | 1,4-di(benzofuran-2-yl)butane-1,4-dione 2-(4-phenoxybenzoyl)benzoic acid | | | | |
| C17H12O7 | 5-(4-methylbenzoyl)benzene-1,2,4-tricarboxylic acid Dalspinin | | | | |
| C20H14O7 | 2-(3,4,5,6-tetrahydroxy-9H-xanthen-9-yl)benzoic acid Ascochytatin | | | | |
| C19H14O4 | 1-(2-methoxybenzoyl)naphthalene-2-carboxylic acid 3,5-Diphenoxybenzoic acid | | | | |
| C17H12O4 | 2-[(2-oxochromen-3-yl)methoxy]benzaldehyde 3-Methylflavone-8-carboxylic acid | | | | |
| C19H14O5 | 3-(6-acetyloxynaphthalen-2-yl)oxybenzoic acid Vulpinic acid | | | | |
| C16H12O5 | 2-(9-oxoxanthen-2-yl)oxypropanoic acid Prunetin | | | |  |
|  |  |  |  |  |  |
| C21H15O5N1 | Dibenzoylmononitrosoorcin 4-(1,3-Dioxo-1,3-dihydro-isoindol-2-ylmethyl)-3-hydroxy- naphthalene-2-carboxylic acid methyl ester | | | | |
| C20H15O5N1 | 2-nitro-4-(3-phenylmethoxyphenyl)benzoic acid | | | |  |
| C18H13O3N1 | 2,6-diphenyl-3-nitrophenol | |  |  |  |
| C21H15O3N1 | cis-4-(p-nitrostyryl)benzophenone | | |  |  |
| C20H15O3N1 | phenyl 3-benzamidobenzoate 3-benzoylamino-4-hydroxybenzophenone | | | | |
| C20H15O6N1 | 7-amino-6-benzoyl-4,9-dimethoxyfuro[3,2-g]chromen-5-one Oxophoebine | | | | |
| C17H13O5N1 | Phthalimidomethyl 3-methoxybenzoate 1-((Ethoxycarbonyl)amino)-4-hydroxyanthraquinone | | | | |
| C17H13O4N1 | aristolamide N-Phthaloyl-L-phenylalanine | | |  |  |
| C15H11O4N1 | 2-(benzoylamino)-α-oxo-Benzeneacetic acid 4'-Nitroflavanone | | | | |
| C15H11O5N1 | 2-(4-acetylphenyl)-4-nitrobenzoic acid Bostrycoidin | | | |  |
|  |  |  |  |  |  |
| C8H8O3S1 | 3-(methylsulfinyl)-Benzoic acid | | |  |  |
| C15H12O6S2 | methyl 2-oxo-2-(4,6,6-trioxo-3,5-dihydro-2H- thiopyrano[3,2-c]thiochromen-3-yl)acetate | | | | |
| C16H12O4S1 | 2-Phenylthiomethyl-3-carboxy-5-hydroxybenzofuran | | | |  |
| C16H12O3S1 | 5-methoxy-3-phenyl-1-benzothiophene-2-carboxylic acid | | | | |
| C15H12O4S1 | 2-benzylsulfanylbenzene-1,3-dicarboxylic acid | | | |  |
|  |  |  |  |  |  |
| **Lipid** |  |  |  |  |  |
| C28H56O2 | Octacosanoic acid. Montanic acid | | |  |  |
| C20H32O2 | Arachidonic acid | |  |  |  |
| C26H52O2 | Hexacosanoic acid | |  |  |  |
| C28H56O3 | 2-Hydroxyoctacosanoic acid | | |  |  |
| C18H36O2 | Stearic acid |  |  |  |  |
| C30H60O2 | Melissic acid |  |  |  |  |
| C28H54O4 | Octacosanedioic acid | |  |  |  |
| C27H54O2 | Heptacosanoic acid | |  |  |  |
| C26H52O3 | 2-Hydroxyhexacosanoic acid | | |  |  |
| C25H50O2 | Pentacosanoic acid | |  |  |  |
|  |  |  |  |  |  |
| C18H30O3S1 | Dodecylbenzenesulfonic acid | | |  |  |
| C16H26O3S1 | 3-(Ethylsulfanyl)-2-[4-(tetrahydro-2-furanyloxy) butylidene]cyclohexanone | | | | |
| C16H26O3S1 | Benzenesulfonic acid, decyl- | | |  |  |
| C12H18O4S2 | 2-[2-(2-methylprop-2-enoyloxy) ethyldisulfanyl]ethyl 2-methylprop-2-enoate | | | | |
| C13H20O4S2 | dipropan-2-yl 2-(1,3-dithian-2-ylidene) propanedioate | | | |  |
|  |  |  |  |  |  |
| **CAS** |  |  |  |  |  |
| C20H10O6 | Bilawsone |  |  |  |  |
| C20H10O5 | Bulgarein |  |  |  |  |
| C21H10O7 | Chembl4163702 | |  |  |  |
| C19H10O5 | Haemodordioxolane | |  |  |  |
| C18H10O5 | Kinobscurinone | |  |  |  |
| C22H12O7 | 4-(9,10-dioxoanthracen-2-yl)oxyphthalic acid | | | |  |
| C22H12O7 | spiroxin C |  |  |  |  |
| C19H10O6 | Dehydrodolineone | |  |  |  |
| C21H10O6 | Eucapsitrione | |  |  |  |
| C21H12O5 | balsaminone A | |  |  |  |
|  |  |  |  |  |  |
| C19H9O5N1 | 3-(1,3-BENZOXAZOL-2-YL) PYRANO[3,2-C]CHROMENE-2,5-DIONE | | | | |
| C18H9O4N1 | Nitrobenzanthracenedione | |  |  |  |
| C17H9O6N1 | 4,11-dihydroxy-5,10-dioxo-1H-naphtho[2,3-f]indole-3-carboxylic acid | | | | |
| C19H11O6N1 | Hypecoumine | |  |  |  |
| C20H11O4N1 | Anthraquinone, 1-(m-nitrophenyl) | | |  |  |
| C17H9O5N1 | 2-Furancarboxylic acid 3-oxo-3H-phenoxazine-7-yl ester | | | |  |
| C20H11O5N1 | Chembl4127273 | |  |  |  |
| C16H9O4N1 | Kalbretorine |  |  |  |  |
| C18H11O4N1 | Atherospermidine | |  |  |  |
| C18H9O6N1 | 6-(2-Nitrophenoxy)-1H,3H-benzo[de]isochromene-1,3-dione | | | | |
|  |  |  |  |  |  |
| C18H8O4S1 | Name:6-hydroxy-4-oxa-11-thia-benzo[def]chrysene-5,12-dione | | | | |
| C17H8O6S1 | 4-Hydroxy-3-(2-thienylcarbonyl)-5H-spiro[furan-2,2'-indene]-1',3',5-trione | | | | |
| C16H8O4S1 | 5,10-Dihydroxyanthra[2,3-b]thiophene-4,11-dione | | | |  |
| C16H8O5S1 | 1-Hydroxypyrene sulfate | |  |  |  |
| C15H10O4S1 | 2-acetyl-8-methoxybenzo[f][1]benzothiole-4,9-dione | | | |  |
| C17H10O4S1 | 7-Oxobenzo[a]phenalene-8-sulfonic acid | | |  |  |
| C18H10O5S1 | 4-(benzenesulfonyl)benzo[f][2]benzofuran-1,3-dione | | | |  |
| C15H10O5S1 | Sulfoflavone |  |  |  |  |
| C16H8O6S1 | 5-hydroxy-7-(3-hydroxy-2-oxochromen-4-yl)-1,3-benzoxathiol-2-one | | | | |
| C20H10O4S1 | Benzo[a]pyrenesulfate | |  |  |  |
|  |  |  |  |  |  |
| C15H7O5N1S1 | 6-Oxo-6H-anthra(9,1-cd)isothiazole-3-carboxylic acid | | | |  |
| C18H9O4N1S1 | bis-6H-1-benzopyrano[4,3-b][1,4]thiazine | | |  |  |
| C16H9O4N1S1 | 4-o-nitrophenylthio-1,2-naphthoquinone | | |  |  |
| C17H9O5N1S1 | 3-(Benzenesulfonyl)benzo[f][1,2]benzoxazole-4,9-dione | | | |  |
| C16H9O3N1S1 | 3-(benzo[d]thiazol-2-yl)-8-hydroxy-2H-chromen-2-one | | | |  |
| C14H7O3N1S1 | 9,10,10-trioxothioxanthene-3-carbonitrile | | | |  |
| C16H9O5N1S1 | 1-(benzenesulfonyl)indole-2,3-dicarboxylic anhydride | | | |  |
| C13H7O5N1S1 | 5-Hydroxy-7-(4-nitrophenyl)-1,3-benzoxathiol-2-one | | | |  |
|  |  |  |  |  |  |
| **Protein** |  |  |  |  |  |
| C19H19O4N1 | Nandinine |  |  |  |  |
| C17H17O6N1 | Maculosine |  |  |  |  |
| C17H17O5N1 | Hippeastrine |  |  |  |  |
| C15H15O5N1 | Actiketal |  |  |  |  |
| C15H15O6N1 | Ascorbigen |  |  |  |  |
| C21H21O4N1 | Zindoxifene |  |  |  |  |
| C17H17O4N1 | Carbobenzoxyphenylalanine | | |  |  |
| C21H21O6N1 | Corynoline |  |  |  |  |
| C21H21O5N1 | Corlumine |  |  |  |  |
| C18H19O5N1 | Melicopicine |  |  |  |  |
|  |  |  |  |  |  |
| **UHC** |  |  |  |  |  |
| C20H28O2 | Retinoic acid |  |  |  |  |
| C20H14O2 | 1,2-Dibenzoylbenzene | |  |  |  |
| C20H16O2 | Triphenylacetic acid | |  |  |  |
| C21H16O2 | Diphenylmethyl benzoate | |  |  |  |
| C22H16O2 | 1,12-Dimethoxyperylene | |  |  |  |
|  |  |  |  |  |  |
| C21H13O2N1 | 4-PHENYLBENZOFURO[3,2-g]QUINOLIN-2(1H)-ONE | | | |  |
| C20H11O2N1 | Anthrachinolinchinon | |  |  |  |
| C20H12O2N2 | 6-(3-Pyridinyl)-5H-indeno(1,2-c)isoquinoline-5,11(6H)-dione | | | | |
| C20H10O2N2 | Dibenzophenazinedione | |  |  |  |
| C20H13O2N1 | Styrylnaphthalimide | |  |  |  |
